# Broad-acting antivirals: the pursuit of pan-viral therapeutics in the era of pandemics

**DOI:** 10.1128/jvi.00077-26

**Published:** 2026-03-23

**Authors:** Ekaterina Bayurova, Dmitry Kostyushev, Andrey Tikhonov, Vladimir Chulanov, Ilya Gordeychuk

**Affiliations:** 1 Chumakov Federal Scientific Center for Research and Development of Immune-and-Biological Products of Russian Academy of Sciences (Institute of Poliomyelitis) 488095 , Moscow, Russia; 2 Department of Biotechnology, Sechenov University 68477, Moscow, Russia; 3 Center for Precision Genetic Technologies for Medicine, Engelhardt Institute of Molecular Biology, Russian Academy of Sciences 68470 , Moscow, Russia; 4 Laboratory of Genetic Technologies in Drug Development, Martsinovsky Institute of Medical Parasitology, Tropical and Vector-Borne Diseases, Sechenov University 68477, Moscow, Russia; 5 Faculty of Bioengineering and Bioinformatics, Lomonosov Moscow State University 274435 , Moscow, Russia; 6 Laboratory of Experimental Therapy of Infectious Diseases, Martsinovsky Institute of Medical Parasitology, Tropical and Vector-Borne Diseases, Sechenov University 68477, Moscow, Russia; 7 People’s Friendship University 64948, Moscow, Russia; 8 Department of Infectious Diseases, Sechenov University 68477, Moscow, Russia; 9 Institute for Translational Medicine and Biotechnology, Sechenov University 68477, Moscow, Russia; New York University Department of Microbiology, New York, New York, USA

**Keywords:** broad-acting antivirals, host-targeting antivirals, host-targeting agents, repurposing drugs, CRISPR-Cas

## Abstract

The ever-present threat of new viral epidemics makes the scientific community relentlessly work on the development of universal methods of antiviral therapy. The development of broad-spectrum antivirals (BSAs) focuses either on substances acting directly on viral proteins (direct-acting antivirals [DAA]) or on substances directed at the cell’s own proteins (host-targeting antivirals [HTA]). Decades of development have led to the market entry of a number of DAAs with a wide range of antiviral activities; however, their clinical approval has been obtained for individual infections. HTAs have a number of advantages over DAAs, such as a wider range of antiviral activities and a high genetic barrier to viral resistance, which is undoubtedly important when preparing for a battle with an unknown pathogen. The COVID-19 pandemic has allowed for multiple clinical trials for repurposed HTAs, previously licensed for the treatment of other diseases, including cancer. Despite the enormous work done, the arsenal of BSAs capable of protecting against future pandemics caused by pathogen X is very limited. In this review, we described data on the most studied DAAs and HTAs, effective against at least two unrelated viral pathogens, focusing on those that have been studied in late preclinical and clinical trials. In the end, we highlighted alternative new approaches such as CRISPR-Cas therapy.

## INTRODUCTION

**Fig 1:**
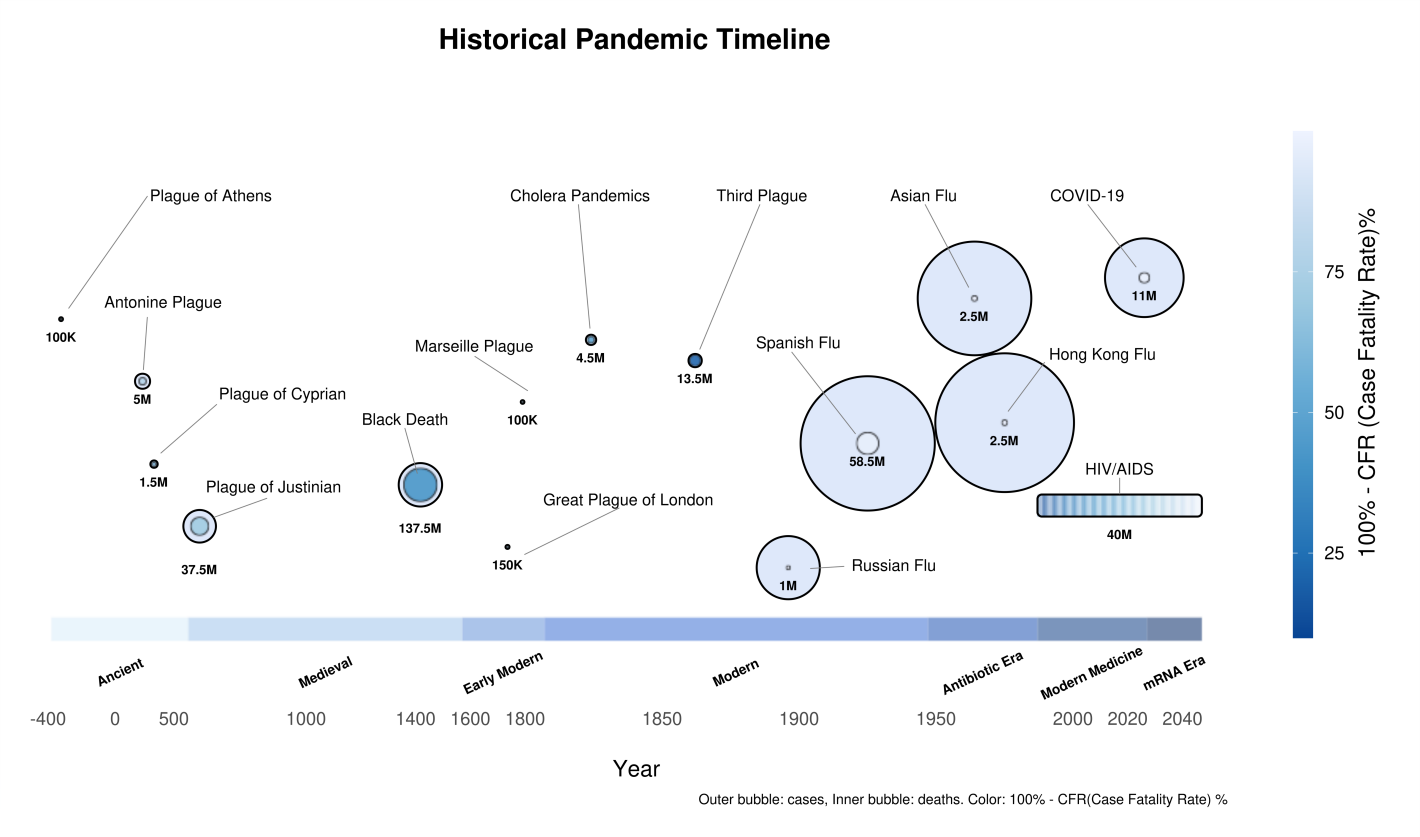
A retrospective timeline of pandemics in human history. The outer bubble indicates the total number of cases, and the inner bubble indicates the number of deaths. The color indicates the case mortality rate (%) from dark blue to light blue.

The World Health Organization (WHO) has documented more than 1,200 outbreaks of epidemic-prone diseases across 188 countries since 2011 (1), underscoring the increasing frequency of health emergencies and the dawn of a pandemic era (2) (Fig. 1). Most high-risk pathogens are zoonotic in origin, unknown to science, and currently circulating among animal species. It is well-established that further human environmental perturbations will likely catalyze the emergence of novel zoonotic viruses (3), with RNA viruses being of particular concern (2). Concurrently, the risk of laboratory-borne infections, resulting from insufficient safety practices, careless handling of biomaterials, or gain-of-function research, cannot be ruled out as a potential source of novel pathogens with pandemic potential.

Current analyses estimate the new pandemic with a probability of 2.5%–3.3% annually, meaning that the chances of another COVID-19-like outbreak occurring within the next 10 years are 22%–28%, and of 47%–57% chance for the pandemic to occur within the next 25 years.

The critical value of a proactive research strategy was demonstrated during the COVID-19 pandemic, where prior long-term studies of coronaviruses directly enabled the accelerated development of vaccines and monoclonal antibodies (4). Recognizing this, the WHO has shifted its priority framework from focusing on individual pathogens to studying entire viral families (5). Such broad preparedness contrasts with developing pathogen-specific therapies, which often lag during rapidly evolving outbreaks. Importantly, this research must be inclusive: any virus family currently considered low risk could trigger a future pandemic due to genetic alterations or environmental shifts (6) and should not be neglected by the scientific community.

The pathogen-specific “one drug-one bug” paradigm is ill-suited for pandemic preparedness due to its inherent limitations, including narrow specificity, protracted development cycles, and prohibitive costs (7, 8). A more promising alternative is the development of broad-spectrum antivirals (BSAs) that target either conserved viral elements or essential host factors. Herein, we define a BSA as a compound with demonstrated activity against at least two unrelated viral pathogens. Despite the field facing significant challenges, this review summarizes the most substantial progress in developing both direct-acting and host-targeting BSAs, including new-generation therapies based on novel molecular tools, with a particular focus on candidates advancing through clinical trials as well as early drug candidates. Finally, we discuss future directions for the BSA field and speculate on how emerging technologies could facilitate the ultimate goal of a universal antiviral therapy.

## METHODS

Our initial search for the current state of art in the broad-spectrum antiviral development was performed in PubMed and Google Scholar using the following search terms: “broad spectrum antiviral” OR “BSA,” “pan-familia antiviral,” “host targeting antiviral” OR “HTA,” and “combination broad acting antivirals” with applied filters on the date of publication “2020-2025.” Data on compounds effective against at least two unrelated pathogens were included in the review. Data on compounds effective against multiple variants of a single virus (i.e., multiple isolates of SARS-CoV-2) were excluded from the search. Based on the results of our initial search, we conducted further literature searches to obtain more detailed descriptions of antiviral activity mechanism(s), experimental confirmation, and clinical application.

Due to the complexity of antiviral research, data on drug activity are inherently difficult to harmonize. Across the revised literature, antiviral assays vary significantly depending on the virus studied. For the purposes of this analysis, a positive *in vitro* result is defined as either the prevention of plaque formation or a significant reduction in viral genome copies measured by PCR, based on the metrics reported in the source studies. Positive *in vivo* activity refers to a demonstrated reduction in virus-induced pathology and/or prevention of viral shedding. A successful clinical outcome is defined according to the endpoints established by the respective research groups. Unless otherwise specified, all described substances were administered immediately after infection.

The relative efficacy of antiviral drugs presented in Figures 3 and 5 is based on reported *in vitro* half-maximal inhibitory concentration (IC₅₀) values under low-dose treatment conditions. It is important to note that this comparative metric is subjective as it is not possible to fully harmonize results across diverse laboratory, preclinical, and clinical studies involving different compounds and viral infection models.

## DIRECT-ACTING BROAD-ACTING ANTIVIRALS

**Fig 2:**
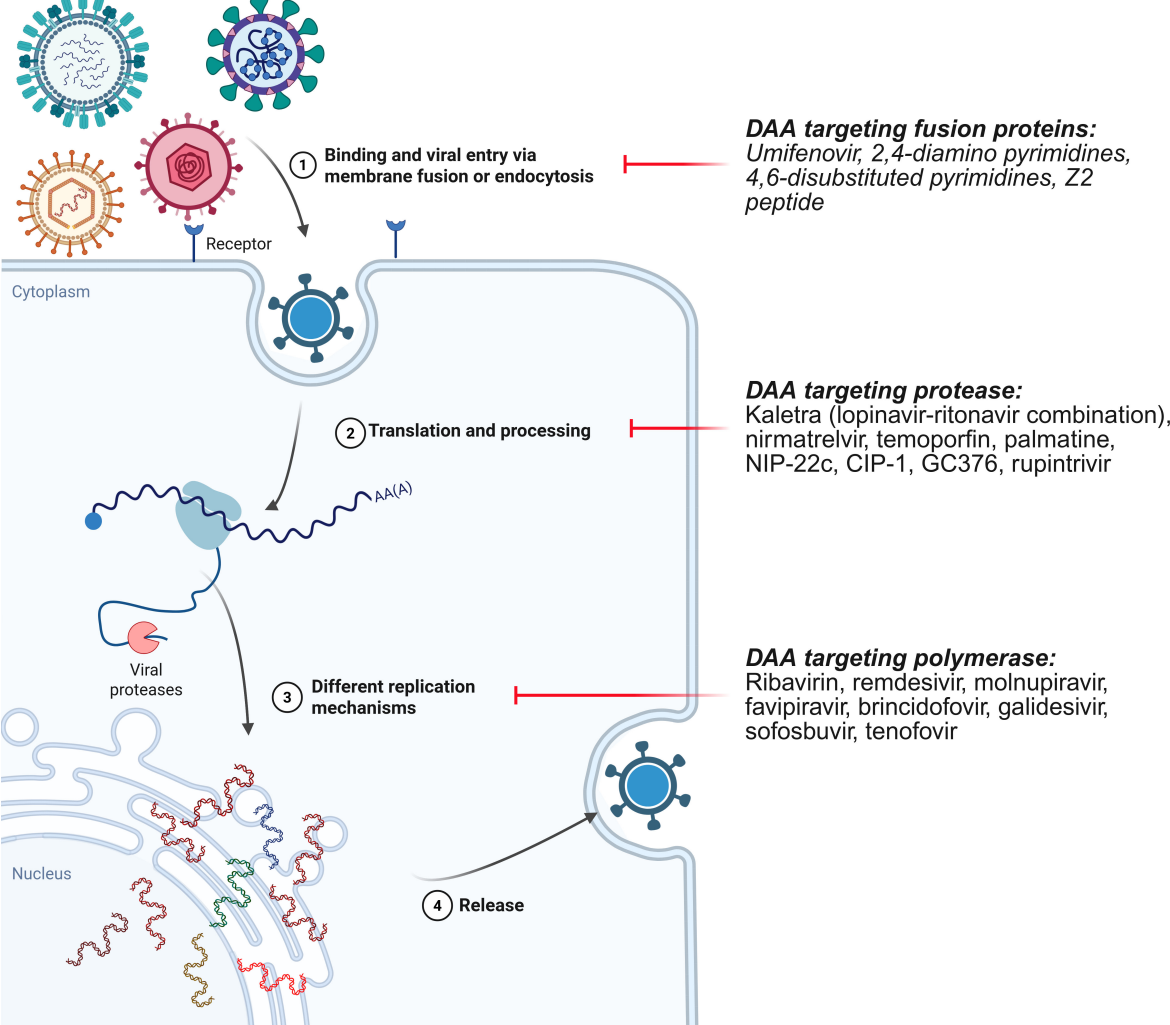
Direct-acting compounds with broad-spectrum antiviral activity. The pictogram illustrates a generic viral life cycle. Classes of inhibitors with broad-spectrum antiviral activity are connected to the specific stages of the viral life cycle they target, with most advancing candidates for each class listed.

**Fig 3:**
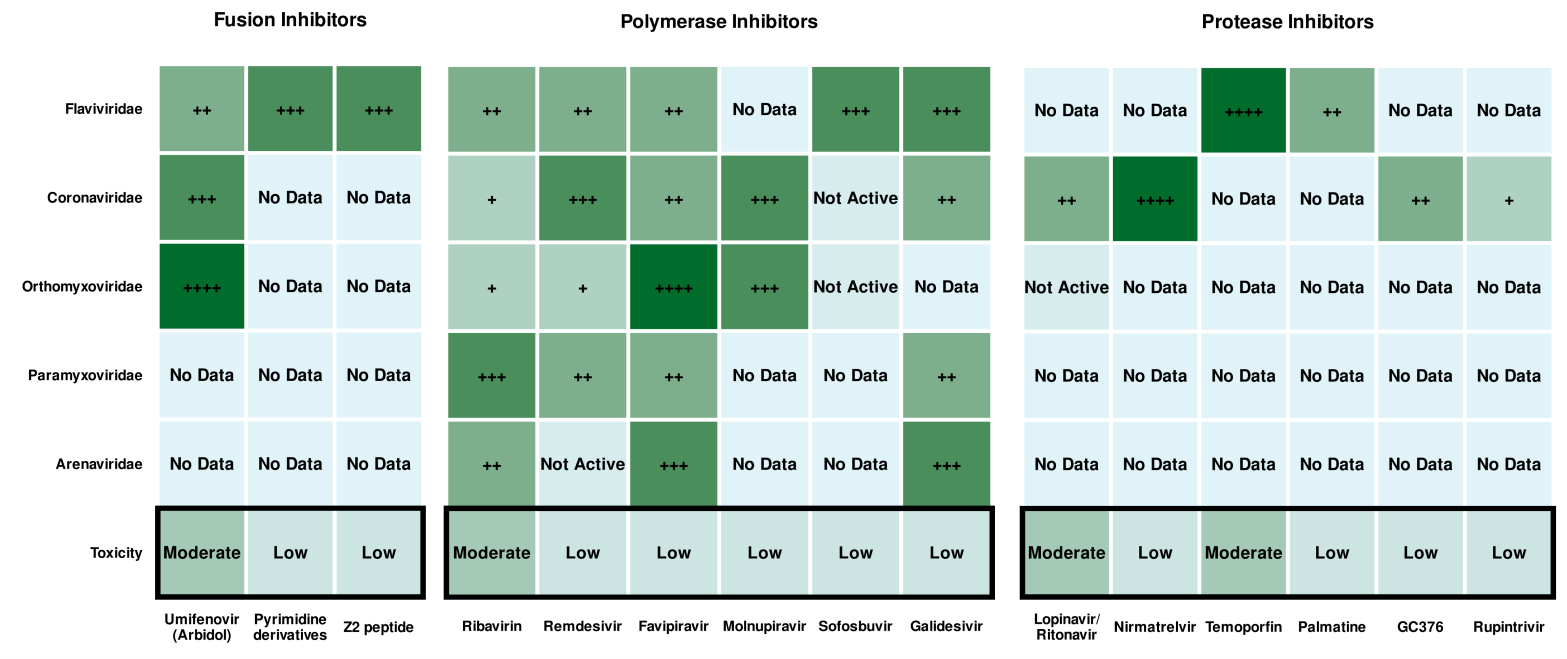
Heat map of the direct-acting and broad-spectrum antiviral compounds’ efficacy among viral families. The color from light green (modest) to dark green (high) and the number of (+) symbols indicate the potency of a particular compound.

Direct-acting antivirals (DAAs) directly target viral components (mostly proteins). The most promising targets for BSAs are the highly conserved enzymes essential for viral replication, such as the polymerase and protease. Separately, drugs targeting viral fusion proteins were developed but present a more complex opportunity as these proteins are among the most virus-specific and are subject to constant mutational pressure. Progress in the development of DAAs is illustrated in Fig. 2 and 3 and in Table S1 and excessively reviewed elsewhere (see, for example, 3, 8), thus being omitted in this review.

Numerous novel DAA compounds can now be identified through structure-based prediction and viral protein similarity analyses (9). However, many of these candidates represent “weak hits,” exhibiting inhibitory concentrations (IC₅₀) that exceed the highest achievable plasma concentration in humans (10). This pharmacokinetic limitation currently precludes their progression to clinical trials or therapeutic use. As discussed later in this review, one promising strategy to overcome this barrier is the implementation of a combinational approach.

A critical limitation in the field is the overwhelming reliance on *in vitro* data, with a stark deficit of robust *in vivo* and clinical evidence. This is reflected in the overall landscape of antiviral drugs: despite decades of research, only approximately 100 antiviral drugs are approved, most targeting just HIV and HCV, and covering only 10 of the over 200 known human pathogenic viruses (8, 11). More than that, finding true pan-viral inhibitors of highly mutable proteins is very elusive. This underscores the need to pivot toward innovative strategies, as well as the development of host-targeting antivirals (HTAs), to achieve true pandemic preparedness.

## HOST-TARGETING BROAD-ACTING ANTIVIRALS

**Fig 4:**
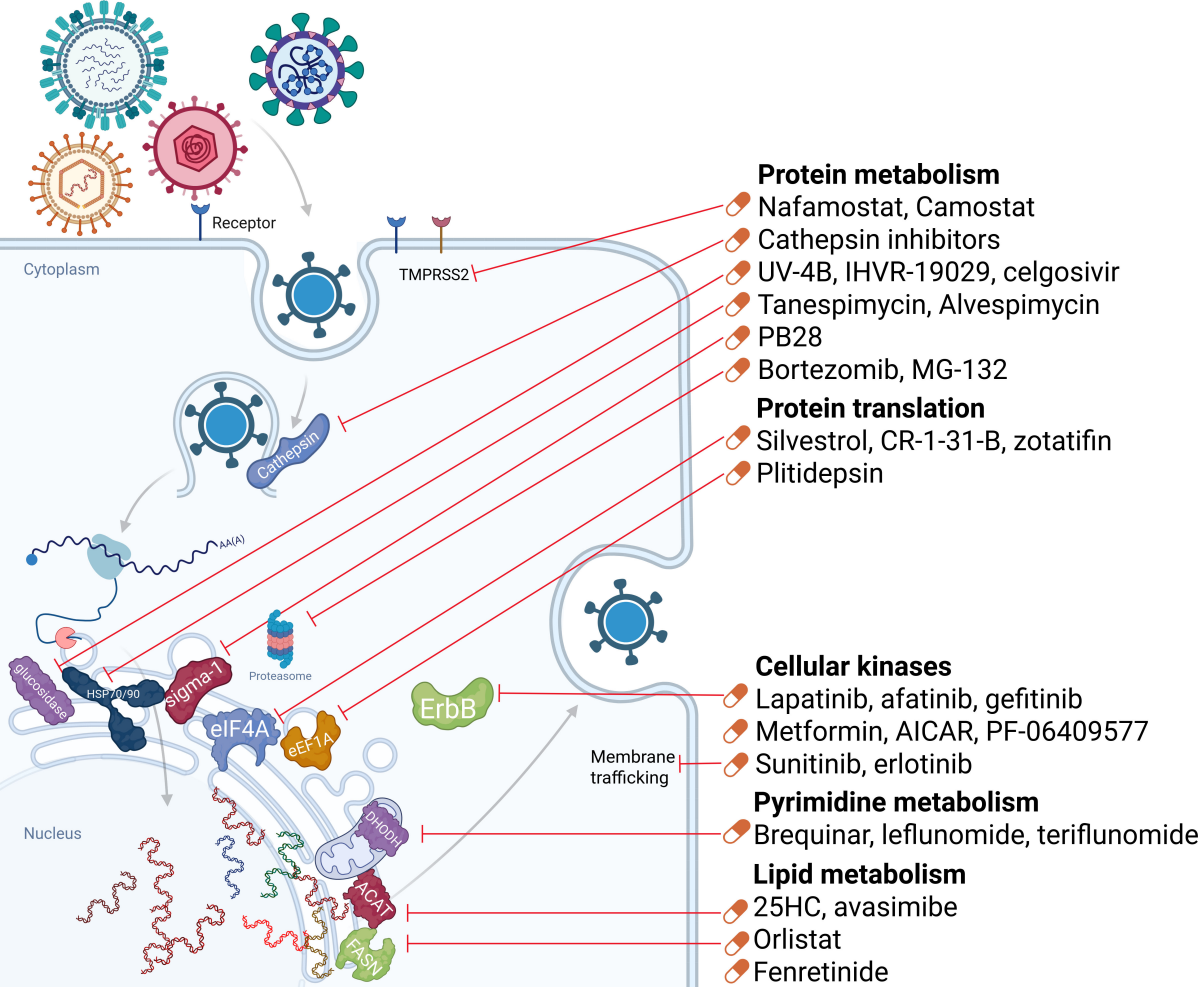
Host-targeting antiviral compounds with broad-spectrum antiviral activity. The pictogram illustrates a generic viral life cycle. Classes of antiviral compounds with broad-spectrum antiviral activity are connected to the specific stages of the viral life cycle they target, with most advancing candidates for each class listed.

The development of DAAs is constrained by their focus on individual viral proteins. Consequently, the pursuit of HTAs, that is, therapies directed to host cellular factors that are hijacked by viruses, represents a more promising avenue for BSA development. This approach leverages the extensive target space of the human proteome. HTAs can be classified based on the affected cellular process: targeting pyrimidine, lipid, or protein metabolism and targeting cellular kinases. Main HTA targets are summarized in Fig. 4. In contrast to DAAs, HTAs can target multiple cellular factors involved in several stages of viral replication. Moreover, some of the extra targets are immunomodulatory, and some are unknown (9). HTAs present a formidable barrier to viral resistance: the likelihood of escape mutations is significantly lower, and the timeframe for their emergence is considerably longer as it requires viruses to circumvent essential host functions rather than mutate a single viral protein (8, 12). The foremost challenge associated with HTAs is their potential for on-target toxicity. Since HTA candidates target human proteins with essential biological functions, any therapeutic intervention can disrupt vital physiological pathways. This mechanism-based toxicity presents a significant barrier to their clinical development and application.

### HTAs targeting lipid metabolism

Cholesterol-25-hydroxylase (CH25H) is an interferon-stimulated gene that produces 25-hydroxycholesterol (25HC) with potent and broad-spectrum antiviral activity. *In vitro* studies have confirmed 25HC’s efficacy against a diverse panel of enveloped viruses, including vesicular stomatitis virus (VSV), HIV, HSV, Ebola virus (EBOV), Rift Valley fever virus (RVFV) and Nipah viruses (13), influenza A (IAV), VZV (14), Zika virus (ZIKV), dengue virus (DENV), yellow fever virus (YFV), West Nile virus (WNV) (15), SARS-CoV, MERS-CoV, and SARS-CoV-2 (16). All reported antiviral activities were associated with blocking viral and cell membrane fusion. Furthermore, antiviral activity against HIV has been validated in a humanized mouse infection model (13) and against ZIKV in both murine and nonhuman primate models (15).

One of the proposed mechanisms of 25HC antiviral activity is inhibition of viral membrane fusion by activating the endoplasmic reticulum (ER)-localized acyl-CoA:cholesterol acyltransferase (ACAT) and subsequent depletion of accessible cholesterol in the plasma membrane (16). Naturally, ACAT inhibitors were also proposed and tested as host-targeting BSAs. Among them, avasimibe was tested in clinical trials for treating atherosclerosis. Although trials were halted due to difficulties in assessing the effects and risks for interactions with other medicines (17), avasimibe is now being repurposed for cancer and antiviral therapy (18). Avasimibe was shown to suppress the replication of SARS-CoV-2 both in ordinary cell cultures and in primary bronchial epithelial cells grown at the air-liquid interface. Avasimibe exerts broad-spectrum antiviral activity by targeting multiple stages of the viral life cycle, including entry, the formation of cytoplasmic replication complexes, and *de novo* particle assembly (19). This has been demonstrated against HCV, HBV, and ZIKV in cell lines and human cerebral organoids (20–23).

Fatty acid synthase (FASN) is a multi-enzyme that is majorly responsible for the *de novo* lipogenesis largely used by viruses. Orlistat is an FDA-approved drug for treating obesity that targets the FASN thioesterase activity (24). Orlistat was shown to affect viral replication organelles and subsequent virion formation of DENV (25), HCV (26), SARS-CoV-2 (27), Coxsackievirus B3 (28), and chikungunya virus (CHIKV) (29). Orlistat injections in SARS-CoV-2 mouse models reduce viral loads and lung pathology, confirming reported *in vitro* antiviral activity (27).

4-Hydroxyphenyl retinamide (fenretinide) is a synthetic analog of retinoic acid that modulates host lipid metabolism with a favorable pre-established safety and pharmacokinetic profile. It was investigated and reported to be effective as an anticancer drug (30). Fenretinide has demonstrated inhibitory activity against multiple viruses, including DENV, WNV, Modoc virus, and HCV, positioning it as a strong candidate for development as a pan-flaviviridae therapeutic (31).

### HTAs targeting pyrimidine metabolism

#### Dihydroorotate dehydrogenase inhibitors

Dihydroorotate dehydrogenase (DHODH) is an enzyme in the *de novo* pyrimidine biosynthetic pathway that catalyzes the dehydrogenation of dihydroorotate to orotate. Many viruses rely on cellular pyrimidine synthesis, thus making DHODH an ideal target for BSA development (32). Brequinar, a compound that was developed back in the mid-1980s as an antimetabolite in cancer and immunosuppression therapies, was recently repurposed for antiviral therapy. Brequinar suppresses the replication of YFV, WNV, Powassan virus, Western equine encephalitis virus, and VSV *in vitro* by affecting viral RNA synthesis (33). Similarly, replication of SARS-CoV-2, ZIKV, and EBOV was reduced by brequinar *in vitro* (34) as well as demonstrating the suppression of rotavirus replication both in cell lines and organoids (35). Brequinar has also been shown to reduce viral titers in a SARS-CoV-2 mouse model when treated in combination with molnupiravir, but not as a monotherapy (36). Phase II clinical trials for COVID-19 treatment reported that brequinar treatment, although safe and well tolerated, failed to reduce viral load and led to prolonged virus shedding (NCT04575038), duplicating results from a mouse model.

Leflunomide is another DHODH inhibitor licensed for arthritis treatment repurposed for antiviral treatment. Leflunomide suppresses respiratory syncytial virus (RSV) replication in cell cultures and reduces viral loads in a cotton rat model even with delayed treatment (until day 3 post-inoculation) (37). Leflunomide suppressed SARS-CoV-2 replication *in vitro*. However, its activity against IAV was low, and effective suppression required treatment with its metabolite, teriflunomide. Teriflunomide was also shown to suppress SARS-CoV-2, ZIKV, EBOV, and IAV replication in cell cultures with effective concentrations in the micromolar range (34).

Of note, leflunomide progressed to clinical trials for COVID-19, where it was reported as safe and well-tolerated but demonstrated no significant impact on clinical outcomes, including symptom prevalence, time to improvement, disease severity, or time to viral clearance from nasal swabs (9). Similarly, teriflunomide failed to recapitulate its *in vitro* efficacy in animal models of SARS-CoV-2 infection (38).

Multiple other DHODH inhibitors have demonstrated broad-spectrum antiviral activity *in vitro*, as reviewed elsewhere (39). Among these, PTC299 (emvododstat) and IMU-838 also entered clinical trials for COVID-19 but likewise failed to show clinical efficacy. The limited *in vivo* and clinical efficacy observed with DHODH inhibitors may be attributed to cellular salvage pathways that can compensate for the depletion of pyrimidine nucleotides (40). This limitation could potentially be overcome through combination therapy with other antiviral agents, as discussed in the following section on combinatorial approaches.

### HTAs targeting protein metabolism

#### α-Glucosidase inhibitors

Iminosugars are BSAs that act as competitive inhibitors of endoplasmic reticulum α-glucosidases I and II, enzymes critical for the proper folding of viral glycoproteins (41). Among them, UV-4B is of special interest now. The iminosugar UV-4B has demonstrated efficacy against IAV and influenza B (IBV) viruses in primary human bronchial epithelial cells and murine lethal models (42). The UV-4B has also demonstrated antiviral activity against DENV in cell cultures, which was further confirmed in mouse models even with delayed initiation of treatment (up to 48 h post-infection) (43). Antiviral activity was also demonstrated *in vitro* against SARS-CoV-2 (44). Obtained results lead to advancement of UV-4B into clinical trials (NCT02061358) that were completed with a good safety and tolerability profile. Nevertheless, product development was halted for business reasons (NCT02696291). Analogously, the related compound celgosivir, with broad antiviral activity *in vitro* (45), was tested in clinical trials against HIV, DENV, and HCV (45). While safe, celgosivir showed no clinical benefit against HIV, HCV (45), or DENV as a monotherapy (46). Furthermore, complete α-glucosidase inhibition by gene knock-out only partially suppressed EBOV and YFV viruses *in vitro*, but the combination of iminosugar IHVR-19029 with favipiravir achieved significant viral inhibition both *in vitro* and *in vivo* (47), highlighting the potential of combination therapies.

#### Furin inhibitors

Furin is a cellular protease essential for cleaving and activating numerous viral surface proteins. Furin inhibitors have been shown to suppress the replication of diverse viruses, including IAV (48), SARS-CoV-2 (49), and MERS-CoV (50). However, a major drawback is furin’s critical physiological role; it processes over 500 human proprotein substrates. Its ubiquitous expression and the lethality of furin knockout in models (51) necessitate caution in developing furin inhibitors, requiring careful management of potential on-target toxicity and autoimmune reactions.

#### Transmembrane Serine Protease 2 inhibitors

Transmembrane Serine Protease 2 (TMPRSS2) is a human protease that is essential for infection by many respiratory viruses. Camostat mesylate and the structurally related drug nafamostat mesylate are TMPRSS2 inhibitors that are already clinically approved in Japan and South Korea for a variety of roles now being repurposed for antiviral therapy (52). Camostat was first found to be effective against IAV *in vivo* in mice, reducing virus secretion (53), and was further confirmed to reduce replication of IAV in primary epithelial cells and reduce subsequent cytokine production (54). Nafamostat was simultaneously confirmed to reduce viral entry of IAV and subsequent inflammatory cytokine production in cells and reduce viral load in mouse lung washes (55). In the COVID-19 pandemic era, camostat and nafamostat were investigated in clinical trials against SARS-CoV-2. Unfortunately, for both drugs, no difference in overall time to recovery or mortality was reported in the list of trials (except one for nafamostat) in hospitalized patients. Camostat treatment was also assessed in nonhospitalized patients with mild-to-moderate COVID-19. No viral load reduction or shortage of symptom resolution period was registered upon several trials (reviewed in 56). However, this forced research of novel TMPRSS2 inhibitors. Among them, MM3122 showed antiviral activity against MERS-CoV and SARS-CoV-2 in cell culture tests, inhibiting viral entry and SARS-CoV-2 cytopathic effect (57). Trypstatin, the other TMPRSS2 inhibitor, prevents cell entry of multiple coronaviruses and influenzaviruses. Trypstatin significantly reduces SARS‐CoV‐2 replication *in vitro* even in the presence of airway mucus and was further confirmed to reduce viral titers and alleviate clinical symptoms in Syrian hamster models (58). These and other (52, 59) novel TMPRSS2 inhibitors have to be evaluated in further trials.

#### Heat Shock Protein inhibitors

Heat Shock Proteins (HSPs), such as HSP70 and HSP90, are molecular chaperones co-opted by viruses to facilitate protein folding and replication (reviewed in 60). Inhibition of HSP70 suppresses orthoflaviviruses, coronaviruses, and orthonairoviruses *in vitro* and *in vivo* (8), though these findings primarily involve tool compounds. Inhibition of HSP90 with geldanamycin or its derivatives (e.g., 17-AAG [tanespimycin] and 17-DMAG [alvespimycin]) has demonstrated *in vitro* activity against a wide range of viruses, including poliovirus (PV), human rhinovirus (HRV), enterovirus A71 (61), Coxsackievirus (62), IAV and IBV (63), RSV (12, 64), HCV (65), CMV (66), EBV (67), HBV (68), HTLV (69), and CHIKV (70). Although these derivatives were evaluated in Phase I/II cancer trials (NCT00089271, NCT00093821, NCT00093405, and NCT00117988), their clinical development was ultimately halted (71). Despite this, the broad-spectrum antiviral activity underscores the potential of HSP inhibition, warranting further investigation with novel compounds.

#### Sigma receptor antagonists

The sigma-1 receptor is an intracellular chaperone involved in cellular stress responses. PB28, a selective sigma-1 receptor antagonist, has recently been reported to exhibit pan-coronavirus activity both *in vitro* and *in vivo* (72), representing a promising new host-targeting strategy.

#### Cathepsin inhibitors

Many viruses enter the cell by endocytosis pathways, ultimately ending in the endolysosomal compartment, where cathepsin and other mechanisms partake in the uncoating and release of viral capsids or genomes. Lysosomal cathepsin L and calpain-1 inhibitors were shown to have pan-coronavirus activity *in vitro* and SARS-CoV-2 RNA reduction in lungs and protection against HCoV-OC43 infection in mouse models (73). At the same time, the role of dozens of cathepsins remains unexplored. As many cathepsin inhibitors are currently developed, targeting single cathepsins or their combinations should be further explored as an approach for preventing and restricting viral infection and spread (74).

#### Proteasome targeting

The development of HTAs *de novo* and the repositioning of existing drugs remain a promising yet challenging area. A case in point is bortezomib, a proteasome inhibitor used in oncology, which has demonstrated broad *in vitro* antiviral activity against SARS-CoV-2, CHIKV, ZIKV, and DENV (75, 76). It acts by interfering with viral protein processing and exploitation of the host proteasomal machinery. Although bortezomib is currently under the proof-of-concept studies as an antiviral, its immunosuppressive and toxic effects could undermine its practical use. At the same time, bortezomib was shown to induce replication and reactivation of many viruses, including HBV (77, 78), VZV (79), and CMV (80).

Bortezomib development as a safe and effective antiviral is hampered by significant challenges, including pro-viral effects under stress conditions, immunomodulatory side effects, and a narrow therapeutic window. This example underscores why, despite numerous clinical trials, the repurposing of such drugs has seen limited clinical success.

MG-132 is another potent, reversible inhibitor of 26S proteasome. MG-132 treatment resulted in improved clinical manifestation of Coxsackievirus B3 (81), reduced CHIKV viral titer in cell culture model (82), suppressed replication of CMV (83), and was shown to suppress HSV-1 replication *in vitro* (84). An important feature of MG-132 is that its inhibitory capacity is not only restricted to the proteasome but also involves cellular calpains, cathepsins, and viral proteases. Due to this, it was capable of suppressing viral entry of SARS-CoV (slightly affected by bortezomib, the selective proteasome inhibitor) (85) and SARS-CoV-2 replication by inhibition of both the main viral protease and cellular cathepsin-L (86). Noteworthily, the study on MG-132 activity against HEV revealed nonspecific antiviral effect occurring in reduction of cellular RNA and proteins (87).

#### Inhibitors of protein translation

Eukaryotic translation initiation factor 4A (eIF4A) is a cap-dependent DEAD-box RNA helicase largely hijacked by RNA-viruses to resolve their 5′-untranslated regions during the initiation of protein synthesis (88). Additionally, translation of many protooncogenes is eIF4A-dependent, which made eIF4A inhibitors perspective anticancer therapeutics now in clinical trials (NCT04092673 and NCT03675893). Rocaglates are a class of translation inhibitors originally isolated from the Aglaia species. Their synthetic analogs have now been developed to enhance potency and bioavailability (89). One of the most studied rocaglates in terms of antiviral activity is the natural compound silvestrol. It was shown to suppress the replication of EBOV (90), ZIKV (91), MERS-CoV, HCoV-229E, and IRES-dependent PV type 1, HRV (92), HEV (93), and CHIKV (94) in cell cultures and exert low cytotoxic effects. Silvestrol also inhibits IAV replication in cell cultures when added early after infection, however with reported cytotoxicity (95). The limitations for large-scale application of silvestrol include the time consumption and sophisticated synthesis, which moved the field to the development of other synthetic rocaglates such as CR-1-31-B and zotatifin (eFT226). Antiviral potency and cytotoxicity of these three rocaglates (namely, zototifin, CR-1-31-B, and silvestrol) were determined *in vitro* using multiple viral 5′-UTRs and a panel of primary and immune cells, with zotatifin reported as the least toxic, but also the least potent (96). CR-1-31-B shared broad antiviral activity with silvestrol and inhibited the replication of HCoV-229E, MERS-CoV, ZIKV, Lassa fever virus (LFV), Crimean-Congo hemorrhagic fever virus, and HEV *in vitro* with nanomolar effective concentrations (97). Zotatifin and CR-1-31-B inhibit the replication of HCoV-229E, MERS-CoV, and SARS-CoV-2 *in vitro* in nanomolar concentrations. However, replication of MERS-CoV in a human airway epithelial cell model was only slightly suppressed by zotatifin. In contrast, CR-1-31-B virtually cleared the virus in nanomolar concentrations (96, 97). Subcutaneous injection of zotatifin treatment was evaluated in mild-to-moderate COVID-19 patients in clinical trials (NCT04632381). It is the first eIF4A inhibitor in the rocaglate family to enter clinical trials as an antiviral, showing safety and good tolerance as well as demonstrating a trend in clinical antiviral activity for mild-to-moderate COVID-19 (98).

Eukaryotic translation elongation factor 1A (eEF1A) is responsible for delivering aminoacyl-tRNA to ribosomes during the elongation step and is often hijacked by viruses for their replication. Plitidepsin is an eEF1A inhibitor licensed in Australia in combination with dexamethasone for multiple myeloma treatment. Plitidepsin was found to inhibit SARS-CoV-2 replication *in vitro* in different cell lines, which was further confirmed in a mouse model (99). Dissecting the plitidepsin antiviral mechanism revealed the suppression of early translation of SARS-CoV-2 proteins, limiting viral RNA genome replication and subsequent viral *de novo* capsid formation. A high-priority finding is that plitidepsin also reduces the translation of cellular mRNAs at concentrations effective for antiviral treatment, yet it has no effect on cell viability. In case of viral infection, inhibitor capacity was focused on viral RNA and protein translation, illustrating selective antiviral activity (100). This resulted in plitidepsin clinical trials for treatment of moderate COVID-19 infection. Plitidepsin was found to be well tolerated and effective in terms of reducing the oxygen therapy duration (101). However, the Phase III trial was discontinued early due to a significant drop in COVID-19 hospitalizations, which limits the interpretability of its results. The antiviral activity of plitidepsin was also confirmed *in vitro* for MERS-CoV, HCV, ZIKV, and HSV-1, all in nanomolar concentrations (100). Importantly, plitidepsin additionally triggers the phosphorylation of the eukaryotic initiation factor 2 subunit alpha (eIF2α) (102), which opens other possible mechanisms of antiviral activity. Notably, plitidepsin was withdrawn from clinical use for myeloma treatment by the European Medicines Agency due to severe side effects. Thus, despite its promising broad-spectrum antiviral activity and positive clinical results, the broad application of plitidepsin warrants caution.

### HTAs targeting cellular kinases

The epidermal growth factor receptor (EGFR/ErbB) family of tyrosine kinases is involved in the replication of numerous viruses. Lapatinib, a pan-ErbB inhibitor approved for cancer treatment, exhibits broad-spectrum antiviral activity against SARS-CoV-2, Venezuelan equine encephalitis virus (VEEV), DENV, EBOV, Marburg virus (MARV), monkeypox virus (103), and HCV (104) at cell culture models. Moreover, its antiviral activity against VEEV and SARS-CoV-2 was confirmed in organoids and a lethal mouse model of VEEV (103). Other pan-ErbB inhibitors, such as afatinib and gefitinib, suppress the replication of LFV (105) and poxviruses (106) in cell culture assays and CMV in guinea pig models (107).

5′-Adenosine monophosphate-activated protein kinase (AMPK) is a central regulator of cellular metabolism. Viruses hijack AMPK to manipulate autophagy, lipid metabolism, and other processes (reviewed in 108). AMPK agonists like metformin and AICAR inhibit replication of ZIKV, DENV (109), HCV (110), HEV (111), and Kaposi’s sarcoma-associated herpesvirus (112) in cell cultures. The selective AMPK activator PF-06409577 also demonstrates inhibitory potential against WNV, ZIKV, and DENV *in vitro* (109). Metformin has been further evaluated in a clinical trial for YFV (NCT04267809), though results are not yet available.

AP2-associated protein kinase 1 and cyclin G-associated kinase regulate endocytosis and Golgi trafficking, processes commonly exploited by viruses. The combination of sunitinib and erlotinib—approved anticancer drugs that inhibit these proteins—shows broad-spectrum antiviral activity against HCV, DENV, EBOV, WNV, ZIKV, CHIKV, Junin virus, and RSV (113).

**Fig 5:**
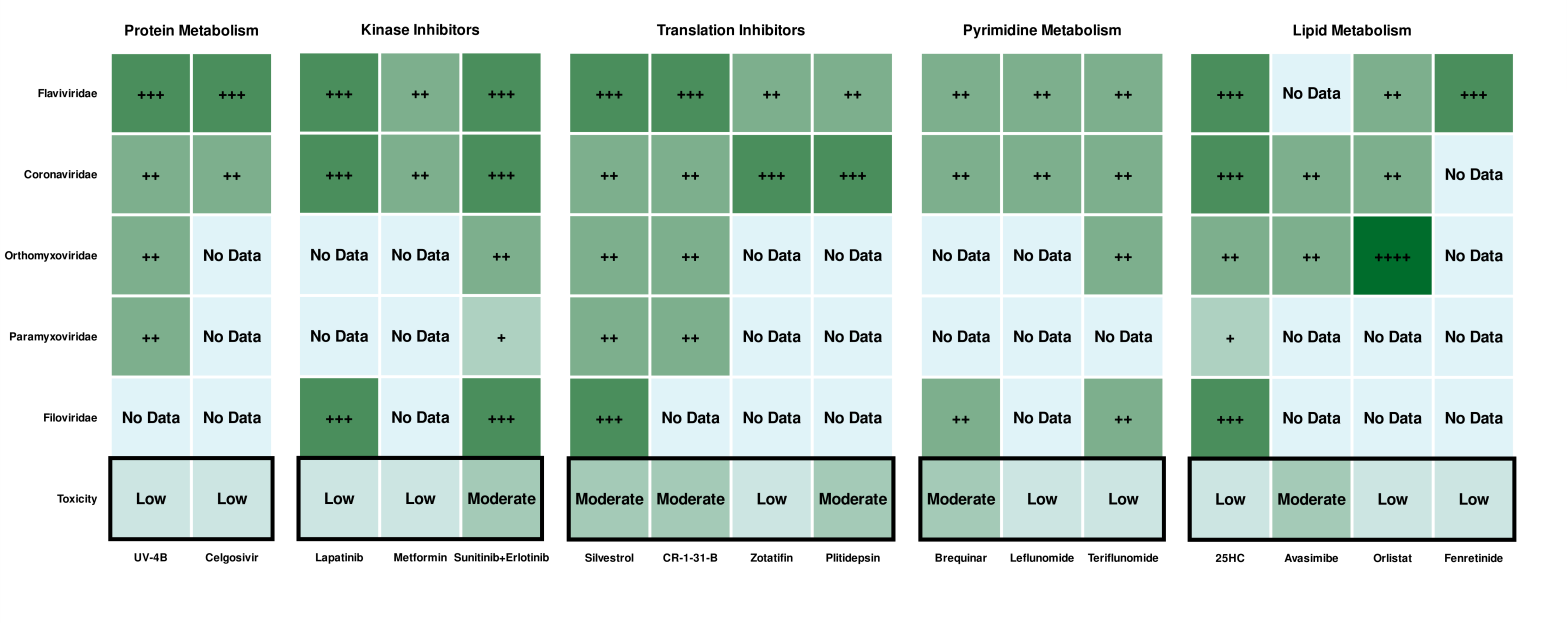
Heat map of the broad-acting host-targeting antiviral compounds’ efficacy among viral families. The color from light green (modest) to dark green (high) and the number of (+) symbols indicate the potency of a particular compound.

The overall activity of the described compounds and their progress in HTA development are summarized in Fig. 5.

HTA development faces significant challenges related to safety and selectivity. Inhibiting critical host proteins can lead to on-target toxicity (114). A potential path forward is to emulate cancer therapeutics, which exploit differential dependence between diseased and healthy cells on specific pathways. For example, cancer cells often exhibit heightened dependence on stress proteins like HSP70 (115). Similarly, inhibitors such as JC40 can suppress DENV replication with minimal host cell toxicity (116). This underscores the need for careful toxicity profiling, akin to standards in oncology.

Additional complexities include the dual nature of some host factors, which may inhibit one virus while enhancing another. For instance, while interferon-induced proteins with tetratricopeptide repeats (IFITs) generally have broad-spectrum antiviral effects, IFIT2 and IFIT3 may exert pro-viral effects during IAV infection (117). Furthermore, host-directed inhibitors may be influenced by host genetic polymorphisms (114), complicating predictable efficacy.

From a positive perspective, HTAs offer a high genetic barrier to resistance as viral evolution cannot easily overcome the inhibition of a host target. This has been demonstrated in studies where no viral escape mutants emerged (12, 118). HTAs can exert their effects through multiple mechanisms. For instance, DHODH inhibitors exhibit at least three distinct modes of action: depletion of the intracellular pyrimidine pool, activation of interferon-stimulated genes (ISGs), and anti-inflammatory activity (39). This multifaceted activity likely underlies their broad-spectrum antiviral profile. HTAs also present a strategy for treating rare viral diseases where developing pathogen-specific antivirals is economically unfeasible, such as JC polyomavirus-induced progressive multifocal leukoencephalopathy (119).

In conclusion, while the development of HTAs is hindered by fundamental biological challenges—with only a few candidates advancing to clinical trials—their potential for broad-spectrum activity and high barrier to resistance underscores their value. Overcoming these limitations will require innovative approaches to ensure selectivity and safety, potentially offering transformative solutions for pandemic preparedness and neglected viral infections.

## COMBINATIONAL THERAPY

Combinational therapy represents a gold standard for treating chronic viral infections such as HCV and HIV and has also demonstrated efficacy against acute viral infections (120–123). This approach is designed to address key limitations of antiviral drugs, including the development of resistance, high therapeutic doses, and associated toxicities. Critically, combination therapy often results in synergistic rather than merely additive effects, where the total therapeutic outcome exceeds the sum of each drug’s individual contribution.

In the context of pandemic preparedness, combinations incorporating BSAs can extend the antiviral range of the constituent drugs (9), shifting the treatment paradigm from “one drug–one bug” toward a “one cocktail to fit it all” strategy. For many antivirals, effective concentrations vary across different viruses, leading to inconsistent efficacy in animal and human studies even at similar serum drug levels. Synergistic effects that allow for reduced effective doses could therefore enhance the real-world utility of these BSAs (124).

In chronic viral infections, combinations typically involve multiple DAAs or DAAs plus IFN, primarily to suppress drug-resistance mutations (125–127). Pharmacokinetic enhancers such as cobicistat and ritonavir are also frequently used in combinations to inhibit the metabolism of the primary agent, permitting lower and safer dosing (128). For pandemic preparedness, combining a DAA with a HTA is often considered preferable (123). Overall, combination therapy is viewed as key to developing potent, clinically effective BSAs (39).

Over 500 antiviral drug cocktails are currently in various stages of development. Among combinations with known targets, most consist solely of DAAs, followed by DAA+HTA and HTA+HTA combinations (9). Notably, clinical studies of antiviral cocktails have predominantly focused on activity against single viruses—mainly HIV, HCV, HBV, SARS-CoV-2, and HSV (9). Even when broad *in vitro* or *in vivo* efficacy has been reported, clinical confirmation of broad-spectrum activity remains a necessary, but unmet need.

The clinical efficacy of HTAs can be limited due to redundant host pathways that compensate for inhibition of a single target, as observed with DHODH inhibitors (39). Combination therapy may overcome this by simultaneously targeting multiple pathways. For instance, the combination of brequinar (a DHODH inhibitor) and an ENT1/2/4 inhibitor like dipyridamole has shown synergistic effects not only in cancer but also against SARS-CoV-2 (129, 130), HSV-1 (131), IAV (132), and RSV (133) in cell-based studies. This combination entered a clinical trial for moderate COVID-19 but was terminated early due to insufficient patient enrollment (NCT05166876), leaving its clinical impact unassessed.

Several platforms exist to predict drug synergy and identify promising combinations among available antivirals. SynergyFinder (https://synergyfinder.fimm.fi/), for example, is a public web tool that analyzes data from checkerboard assays to quantify synergistic interactions. Other scoring systems have also been proposed (9). However, all computational predictions require thorough validation in both *in vitro* and *in vivo* models.

In summary, while combination therapy based on broad-spectrum antivirals is widely regarded as an ideal strategy for pandemic preparedness, no clinically validated panel of such cocktails currently exists.

## FUTURE PERSPECTIVE

The development of gene therapy in recent years has led to the approval and market entry of a number of gene therapy drugs (134). Aside from drugs based on autologous T cells used for the treatment of cancer, a number of drugs based on viral vectors used for the treatment of genetic and viral diseases have been approved.

Two approved gene therapies are among those with high interest in the context of this review. Nadofaragene firadenovec-vncg (Adstiladrin, Ferring Pharmaceuticals A/S) is a nonreplicating adenoviral vector-based gene therapy encoding for interferon-α2b (135). This is a milestone therapy indication that gene therapy coding for cytokines is safe and efficient in humans. The other is exagamglogene autotemcel (exa-cel, CASGEVY, Vertex Pharmaceuticals Incorporated), a first nonviral cell therapy designed to reactivate fetal hemoglobin synthesis through *ex vivo* CRISPR-Cas9 gene editing (136). This is a milestone that opens the way for other therapies based on CRISPR-Cas technology.

CRISPR-Cas is widely used to dissect mechanisms of antiviral immunity and to identify novel antiviral genes (reviewed in 137). Substantial experimental and preclinical data demonstrate the use of CRISPR-Cas for inactivating viral genomes, with many approaches showing efficacy in clearing infections within days of delivering Cas/sgRNA complexes (138–140). Despite being sequence-specific, CRISPR-Cas systems can be adapted to target conserved regions within a genus or even entire virus families. A promising application is a composite medication comprising multiple Cas/sgRNA variants, each targeting a different virus within a family. This could form the basis for a universal antiviral drug capable of rapidly eliminating viruses from the body. The foundation for such a therapy would require the design of family- or genus-targeting Cas/sgRNA complexes and an effective delivery vehicle, a significant challenge in itself (141, 142). Additional obstacles include achieving tissue and organ specificity, overcoming biological delivery barriers, and navigating virus infection-associated barriers, all of which must be addressed to ensure therapeutic efficacy. Timothy R. Abbott and colleagues recently reported the development of a CRISPR-Cas13d system with predicted pan-coronaviridae and pan-influenzavirus activity targeting conserved regions among viral genomes. Importantly, broad-spectrum antiviral activity was only predicted using a bioinformatics approach (143). Although the reported PAC-MAN system was able to suppress the translation of SARS-CoV-2 regions and suppress IAV infection (both the number of infected cells and the level of gene expression), these results are the very preliminary proof-of-concept study and have to be exhaustively validated. The other way is a CRISPR-activation-based approach (CRISPRa) to induce the expression of antiviral host genes (144, 145) and result in reactivation of latent proviruses or direct suppression of viral genomes by the use of such systems as CRISPRoff (146). CRISPRa activation of genes with antiretroviral activity, including APOBEC3B and APOBEC3G cytidine deaminases, was also shown to suppress HIV replication (147). The main drawback of APOBEC/AID activation is induced mutagenesis in cancer-related genes. However, coupling CRISPRa with attenuated sgRNA technology led to preserved antiviral activity accompanied by precisely controlled APOBEC/AID activation with elimination off-site mutagenesis (144). The presented data pave the way for the development of CRISPR-Cas-based host-targeting broad-acting antiviral drugs.

It is important to note that the documented spectrum of antiviral activities for many of the drugs discussed here is likely narrower than their true potential. A well-known publication bias favors positive results, making it difficult to determine whether a reported lack of activity reflects a genuine biological limitation or simply a lack of testing. For small molecules, public databases such as ChemBank (148) and ChEMBL (149) have been developed to consolidate and standardize research data, with ChemBank notably including screening results even when negative. Similar curated repositories are critically needed for other classes of antiviral agents.

Most data presented in this Review derive from *in vitro* studies, highlighting the so-called “valley of death” in drug development: a prolific discovery phase often leads to little clinical progress. Although strategies to bridge this gap have been proposed (e.g., [150, 151]), the enormous cost of development underscores the urgent need for robust drug-prioritization mechanisms. Compounding this challenge is the poor correlation between *in vitro* antiviral potency in human cell lines and efficacy in complex *in vivo* models. Discrepancies between human and animal target proteins can further undermine predictability, and a study by Hackam and Redelmeier noted that only about one-third of highly cited animal studies translate to human randomized trials—a translation rate not predicted by methodological quality (152). Moreover, insufficient pharmacokinetic data in animal models can lead to inappropriate dosing regimens and false-negative outcomes.

The clinical trial phase remains the most costly and resource-intensive stage, often reliant on private investments (151). Consequently, clinical studies tend to focus on one or two high-profile pathogens (e.g., SARS-CoV-2, EBOV, and HIV). The combined effect of these limitations is that, despite pandemic preparedness efforts, we possess only a limited set of “potentially broad-spectrum antivirals” whose real-world effectiveness remains largely unconfirmed.

## CONCLUSION

Recent viral outbreaks, epidemics, and the COVID-19 pandemic demonstrate a growing threat from novel viruses. Developing virus-specific antivirals is a slow, difficult process with a low market success rate, creating an urgent need for universal antiviral solutions. Current research primarily focuses on two strategies: direct-acting antivirals and host-targeting antivirals.

Many publications describe the development of broad-spectrum DAAs; however, most are based on *in vitro* experiments. This is limited primarily by the lack of relevant animal models for viral infections. Developing such models is necessary to increase the translational potential of this research.

The development of new HTAs and the repurposing of existing drugs from tumor therapy is a promising area, though it has so far seen limited success, largely accelerated by the COVID-19 pandemic. Although multiple clinical trials have evaluated the antiviral efficacy of repurposed drugs, none have been approved for antiviral use. Because host targets have their own biological functions, inhibiting them raises selectivity and toxicity concerns, similar to those in cancer therapy. Furthermore, only a limited number of these drugs have been clinically tested against more than one viral infection or viral family. Thus, despite their proposed broad-spectrum potential, no clinical confirmation has been achieved yet.

Novel challenges demand novel solutions. Advances in gene therapy are paving the way for a new class of antiviral technologies. The focus must now shift toward strategies that are independent of the virus itself and do not rely on inhibiting host factors, but instead on inducing the body’s innate antiviral defenses with a particular focus on the safety of such therapeutics. Potential broad-acting antivirals can be envisioned from the novel and emerging technologies, such as CRISPR-Cas-based, mRNA-based, and protein therapeutics. While data in this area remain limited, indicating a long road ahead, the preliminary research marks a promising beginning.

## Data Availability

No new data were created or analyzed in the article.

## SUPPLEMENTAL MATERIAL

Table S1 jvi.00077-26-s0001.docx Comparative characteristics of direct-acting and broad-spectrum antiviral drugs targeting various viral proteins: pros, cons, and clinical status of development.

ASM does not own the copyrights to Supplemental Material that may be linked to, or accessed through, an article. The authors have granted ASM a non-exclusive, world-wide license to publish the Supplemental Material files. Please contact the corresponding author directly for reuse.
