# Supplementary material for: Broad-acting antivirals: the pursuit of pan-viral therapeutics in the era of pandemics
Source: J Virol. 2026 Mar 23;100(5):e00077-26. doi: 10.1128/jvi.00077-26 (PMC13185641; doi:10.1128/jvi.00077-26)
Supplement: Table S1 — Comparative characteristics of direct-acting and broad-spectrum antiviral drugs targeting various viral proteins: pros, cons, and clinical status of development. [file jvi.00077-26-s0001.docx]

Supplementary Table S1. Comparative characteristics of direct-acting and broad-spectrum antiviral drugs targeting various viral proteins: pros, cons, clinical status of development. DENV – Dengue virus; ZIKV – Zika virus; EBOV – Ebola virus; MARV – Marbourg virus; CHIKV – Chikungunya virus; WNV – West Nile virus; JEV – Japanese encephalitis virus; IAV – influenza A virus.

| Antiviral target | Pros | Cons | Viral Families Treated | Clinical Status | References |
| --- | --- | --- | --- | --- | --- |
| **Polymerase Inhibitors** | • High specificity  • Proven clinical efficacy (HCV >90% cure rate)  • Broad-spectrum potential  • Well-established development pathway | • Drug resistance development  • Limited clinical success for many viruses  • Variable efficacy across viral families  • High development costs | • Flaviviridae (HCV, DENV, ZIKV)  • Coronaviridae (SARS-CoV-2)  • Filoviridae (EBOV, MARV)  • Togaviridae (CHIKV) | Approved (HCV);  Phase II/III (others) | ^1–5^ |
| **Protease Inhibitors** | • Proven clinical success (HIV, HCV)  • High potency  • Established regulatory pathways  • Good oral bioavailability | • Structural diversity limits broad-spectrum activity  • Resistance development  • Limited cross-viral family activity | • Retroviridae (HIV)  • Flaviviridae (HCV)  • Coronaviridae (SARS-CoV-2, MERS-CoV)  • Picornaviridae | Approved (HIV, HCV);  Phase II/III (others) | ^1,6–9^ |
| **Fusion Inhibitors** | • Targets essential viral entry step  • Some broad-spectrum activity demonstrated  • Prevents membrane fusion | • High variability of fusion proteins  • Challenging broad-spectrum development  • Virus-specific design needed | • Flaviviridae (DENV, ZIKV, WNV, JEV)  • Orthomyxoviridae (Influenza)  • Coronaviridae | Approved (IAV)  Preclinical/Phase I | ^1,10–12^ |

References

1. Karim M, Lo CW, Einav S. Preparing for the next viral threat with broad-spectrum antivirals. *Journal of Clinical Investigation*. 2023;133(11):e170236.

2. Lamb YN. Remdesivir: First Approval. *Drugs*. 2020;80(13):1355-1363.

3. Beaucourt S, Vignuzzi M. Ribavirin: a drug active against many viruses with multiple effects on virus replication and propagation. Molecular basis of ribavirin resistance. *Current Opinion in Virology*. 2014;8:10-15.

4. Warren TK, Wells J, Panchal RG, et al. Protection against filovirus diseases by a novel broad-spectrum nucleoside analogue BCX4430. *Nature*. 2014;508(7496):402-405.

5. Mulangu S, Dodd LE, Davey RT, et al. A Randomized, Controlled Trial of Ebola Virus Disease Therapeutics. *N Engl J Med*. 2019;381(24):2293-2303.

6. Hashemian SMR, Sheida A, Taghizadieh M, et al. Paxlovid (Nirmatrelvir/Ritonavir): A new approach to Covid-19 therapy? *Biomedicine & Pharmacotherapy*. 2023;162:114367.

7. Chan SW. Current and Future Direct-Acting Antivirals Against COVID-19. *Front Microbiol*. 2020;11:587944.

8. Kim Y, Lovell S, Tiew KC, et al. Broad-Spectrum Antivirals against 3C or 3C-Like Proteases of Picornaviruses, Noroviruses, and Coronaviruses. *J Virol*. 2012;86(21):11754-11762.

9. Li Z, Brecher M, Deng YQ, et al. Existing drugs as broad-spectrum and potent inhibitors for Zika virus by targeting NS2B-NS3 interaction. *Cell Res*. 2017;27(8):1046-1064.

10. De Wispelaere M, Lian W, Potisopon S, et al. Inhibition of Flaviviruses by Targeting a Conserved Pocket on the Viral Envelope Protein. *Cell Chemical Biology*. 2018;25(8):1006-1016.e8.

11. Yu Y, Deng YQ, Zou P, et al. A peptide-based viral inactivator inhibits Zika virus infection in pregnant mice and fetuses. *Nat Commun*. 2017;8(1):15672.

12. Pshenichnaya NY, Bulgakova VA, Lvov NI, et al. Clinical efficacy of umifenovir in influenza and ARVI (study ARBITR). *Terapevticheskii arkhiv*. 2019;91(3):56-63.
